# A Novel Integrative Processing Technology for the Preparation of Rehmanniae Radix Slices

**DOI:** 10.1155/2018/4524797

**Published:** 2018-12-20

**Authors:** Bei-hua Bao, Yan Qian, Fang-fang Cheng, Pei-dong Chen, Yu-dan Cao, Sheng Yu, Ming-qiu Shan, Wei-feng Yao, An-wei Ding, Li Zhang

**Affiliations:** ^1^School of Pharmacy, Nanjing University of Chinese Medicine, Nanjing 210023, China; ^2^Jiangsu Collaborative Innovation Center of Chinese Medicinal Resources Industrialization, National and Local Collaborative Engineering Center of Chinese Medicinal Resources Industrialization and Formulae Innovative Medicine, Nanjing University of Chinese Medicine, Nanjing 210023, China

## Abstract

The traditional processing method for the slices preparation of Rehmanniae roots is time- and energy-consuming and is prone to result in loss of active components during twice water-treatment (once for wash and the other for softening) and drying steps. In this study, we firstly explored an integrative processing technique for Rehmanniae Radix by 2x3 factorial experiment based on the contents of catalpol and verbascoside as measured by HPLC. The potential differences between the traditional stepwise processing technique and the integrative processing technique for catalpol and verbascoside in the prepared slices were investigated. To further confirm the effectiveness of drugs using the integrative processing technique, some pharmacological variables, such as rectal temperature, hematologic parameters (RBC, HGB, HCT, and blood viscosity), and coagulation parameters (TT, APTT, PT and FIB), were detected in a blood-heat and hemorrhage syndrome rat model. Two-way ANOVA analysis showed that drying for 18 h at 50°C was considered as the best combination of process conditions. The mean catalpol and verbascoside contents in the integrative method-processed samples (4.30% and 0.33%, respectively) were higher than those in the traditional method-processed samples (2.61% and 0.21%, respectively). Significant increases in rectal temperature, and hematologic parameters, TT, APTT, and FIB, were observed in the model group rats, compared to the blank group animals (*P*<0.01). Both in the integrative groups and traditional groups, the extracts caused significant decreases in rectal temperature, RBC, HGB, and HCT with increased concentration compared to the model group animals. All coagulation parameters tested were shortened in model rats received two kind prepared slices. There were no significant therapeutic differences between the integrative and the traditional method-processed slices on the hemostasis and hemorheological parameters in this blood-heat and hemorrhage syndrome rat model, indicating that our integrative method may be a feasible technique for processing Rehmanniae Radix slices.

## 1. Introduction

Rehmannia Radix, also known as Dihuang, comes from the fresh or dried root tubers of* Rehmannia glutinosa* Libosch, a plant of the Scrophulariaceae family, and has been used in clinical practice more than 3000 years in China to reduce fever, improve blood circulation, tonify the kidney, and treat osteoporosis and Yin deficiency syndrome [1–8]. Traditionally, the Rehmannia Radix slices, also known as Sheng Dihuang, have been known to affect hemorheology and hemostasis.

‘Páozhì,' the processing of Chinese herbal medicine, is a unique and essential pharmaceutical technology by which the prepared slices are obtained from crude drugs for clinical use following the theory of Traditional Chinese Medicine. In general, the fresh Rehmannia roots are collected in autumn, removed from root stock, rootlet, and soil, and then baked for 3 days at suitable temperature to obtain the unprocessed Rehmanniae root. Then the above unprocessed Rehmanniae roots are softened for 1 day, cut into thick slices, and dried for about 1 day to obtain the Rehmannia Radix slices, i.e., Sheng Dihuang. The traditional processing method from fresh Rehmanniae Radix to the prepared slices is complex and burdensome due to twice water-treatment (once for wash and the other for softening) and drying steps and therefore is prone to result in the loss of active components, with time-consuming and high-cost features.

As the traditional processing technique currently cannot meet the requirements of green production mode, an integrative processing technique has been put forward as an alternative. The integrative processing of Chinese medicinal materials and their prepared slices in the producing area is expected to effectively avoid duplicate steps during processing from medicinal materials to their prepared slices, and consequently the relevant losses and production costs will be reduced and the production efficiency will be improved without compromising the quality of prepared slices, which represents the trend of future development of Páozhì.

Catalpol and verbascoside (acteoside), the most important components of Rehmannia Radix, have many biological effects [9–14] and have been identified as the markers for quality control of Rehmannia Radix in the Chinese Pharmacopoeia (ChP 2015) [1]. Some reports have studied the changes in the chemical components of crude and processed Rehmanniae Radix [15, 16]. The content of catalpol was highest in the fresh Rehmannia roots and decreased over time during processing [17, 18]. Moreover, other reports have studied the metabolism and pharmacokinetics of catalpol and acteoside in rats [19, 20].

Therefore, this study aimed to develop a simple and convenient integrative processing technique to obtain the qualified Rehmanniae Radix slices.

## 2. Materials and Methods

### 2.1. Materials and Reagents

Fresh Rehmanniae Radix was purchased from Dafeng Town, Henan Province of China, and identified by Dr. Qinan Wu (professor from Traditional Chinese Drug Identification Office, Nanjing University of Chinese Medicine, Nanjing, China) as the fresh root tuber of* Rehmannia glutinosa* Libosch., a plant of the Scrophulariaceae family. Verbascoside (Lot 111530-201411) and catalpol (Lot 110808-201210) standards were purchased from National Institutes for Food and Drug Control (Beijing, China). Methanol (for HPLC) and acetonitrile (for HPLC) were purchased from Merck (Darmstadt, Germany). Acetic acid was purchased from Shanghai Shenbo Chemical Co., Ltd. (Shanghai, China). Ethanol absolute, croscarmellose sodium (CMC-Na), and EDTA were purchased from Sinopharm Chemical Reagent Co., Ltd. (Shanghai, China). Sodium citrate was purchased from Tianjin Kemiou Chemical Reagent Co., Ltd. (Tianjin, China). Yunnan Baiyao was purchased from Yunnan Baiyao Group Co., Ltd. (Kuning, China). All kits for measuring thrombin time (TT), fibrinogen (FIB), prothrombin time (PT), and activated partial thromboplastin time (APTT) were purchased from Beijing Steellex Scientific Instrument Company (Beijing, China).

### 2.2. Animals

Male SPF Sprague-Dawley rats weighing between 180-220 g were obtained from the laboratory animal center of Zhejiang Province, China, Certificate No. SCXK (Zhe) 2014-0001.

### 2.3. Apparatus and Instruments

The major apparatus and instruments used in the study are as follow: Agilent Technologies 1290 Infinity high-performance liquid chromatograph equipped with a quaternary pump, a autosampler, and a DAD detector (Agilent Technologies, USA), Electronic balance (METLER, MS-105DU), Milli-Q Ultra-pure water system (Milli-Q DIRECT8, USA), Electronic Thermometer (OMRON MC-347), Hemorheological Analyser (Beijing Gtmsteellex Science Instrument Co., Ltd), Fully Automatic Hemocyte Analyzer (Nihon Kohden Corporation, Japan, MEK~6318K), Platelet Aggregation and Coagulation Factor Analyzer (Beijing Steellex Scientific Instrument Company, LG-PABER), and centrifuge (ShangHai Anting Scientific Instrument Factory).

### 2.4. Optimization of Integrative Processing Technique

According to the processing requirements for Rehmanniae Radix described in ChP 2015 and based on the preliminary experiment results, the drying time and drying temperature were identified as main factors affecting the quality of Rehmanniae Radix. A preliminary 2x3 full factorial experiment was conducted to optimize and determine the adequate levels for each factor. The contents of catalpol and verbascoside were quantitatively determined according to the assay method of catalpol and verbascoside in Rehmanniae Radix described in ChP 2015 [1]. The selected factors and levels are shown in Table 1.

### 2.5. Sample Preparation

#### 2.5.1. Preparation of Integrative Method-Processed Samples

The cleaned fresh Rehmanniae Radix materials were directly cut into thick slices (5 mm) and baked for 18 hours at 50°C to obtain the integrative method-processed slices.

#### 2.5.2. Preparation of Traditional Method-Processed Samples

The cleaned fresh Rehmanniae Radix materials were baked for 3 days at 40°C to obtain the unprocessed Rehmannia roots. The unprocessed Rehmannia roots were softened for 1 day, cut into thick slices (5 mm), and then dried for 16 hours at 60°C.

### 2.6. HPLC Analysis of Catalpol and Verbascoside

The reference standard solutions of catalpol and verbascoside were prepared at a concentration of 0.1 mg/mL in mobile phase. A quantity of Rehmanniae roots were cut into small pieces (about 5 mm × 5 mm), dried under reduced pressure at 80°C for 24 hours and ground into coarse powder. Approximately 0.8 g of coarse powder was accurately weighed and transferred into an erlenmeyer flask with cap. Then 50 mL methanol was added. The total weight of this erlenmeyer flask was recorded, and the solution was extracted by refluxing for 1.5 h in a hot water bath, then weighed again after cooling. An appropriate amount of methanol was added to compensate the loss weight during refluxing extraction, shaken and filtered. The initial filtrates were discarded, and an aliquot of 10 mL subsequent filtrates were transferred into an evaporating dish and concentrated to almost dry in a water bath. The concentrated residues were dissolved with mobile phase and the resulting solution was transferred into a 10 mL volumetric flask and diluted to volume with mobile phase, and then filtered through a 0.22 *μ*m microfiltration membrane after shaking for further use. For analysis of catalpol, 10 *μ*L of the above filtrate and catalpol reference solution were subjected to HPLC. HPLC analysis of catalpol was performed on a Kromasil C_18_ Column (250 mm × 4.6 mm, 5 *μ*m) with acetonitrile-0.1% phosphoric acid solution (1:99, v/v) as mobile phase and the detection wavelength was at 210 nm. For analysis of verbascoside, 20 mL of the above filtrates was pipetted into an appropriate container and concentrated to almost dry under reduced pressure to remove solvent. The concentrated residues were dissolved with mobile phase. The resulting solution was transferred into a 5 mL volumetric flask and diluted to volume with mobile phase and then filtered through a 0.22 *μ*m microfiltration membrane after shaking. Finally, 10 *μ*L of the obtained filtrate and verbascoside reference solution were subjected to HPLC. HPLC analysis of acteoside was performed on a Kromasil C_18_ Column (250 mm × 4.6 mm, 5 *μ*m) with acetonitrile-0.1% acetic acid solution (16:84, v/v) as mobile phase and the detection wavelength was at 334 nm.

### 2.7. Pharmacological Effects

#### 2.7.1. Preparation of Test Solutions

The dose formulations of integrative method-processed and traditional method-processed slices of Rehmanniae Radix were prepared as a suspension in 0.5% CMC-Na solution at the required concentrations of 0.15, 0.3, and 0.6 g/mL, respectively. Yunnan Baiyao was used as the active comparator and formulated as a suspension in 0.5% CMC-Na solution at the concentration of 0.05 g/mL. Dried ginger extract was obtained as followed. Adequate amount of prepared dried ginger slices was soaked with 8-fold of water for 1 h, then boiled for 1 h, and filtered to gain the filtrate. Repeat the above steps three times. All filtrates were collected and concentrated to obtain the ginger extract of 1.5 kg/L.

#### 2.7.2. Group Assignment and Treatment

Ninety Sprague-Dawley rats were assigned randomly into one of 9 groups (n=10 in each group): the blank group, the model group, the active comparator group, low-, medium-, and high-dose integrative groups, and low-, medium-, and high-dose traditional groups. All rats were provided with 5% liquor replacing water, except for rats in the blank group which were provided with water ad libitum. At 8:30 am, all rats except for the blank group were orally given the dried ginger extract at 10 mL/kg body weight daily, and the blank control group animals were orally given the same volume distilled water. After 7 h, the animals in the active treatment group received Yunnan Baiyao suspension (0.5 g/kg); the animals from all integrative groups and traditional groups received 1.5, 3, and 6 g/kg test solution as the corresponding low, medium, or high doses, respectively, and the animals in the blank control group and the model group received the same volume 0.5% CMC-Na; all animals were dosed at 10 mL/kg body weight. Dried ginger extract, Yunnan Baiyao suspension, and test solutions were administered for 14 days. Animal welfare and experimental procedures strictly adhered to the Guide for the Care and Use of Laboratory Animal and were approved by the Animal Ethics Committee of the Nanjing University of Chinese Medicine.

#### 2.7.3. Rectal Temperature

The rectal temperature of all animals was measured on days 13 and 14 after modeling, and the changes in rectal temperature before and after modeling were determined.

#### 2.7.4. Hemorheological Measurements

At 40 min after administration on day 14, blood samples for measurement of red blood cell (RBC) count, hemoglobin level (HGB) and hematocrit (HCT) were taken from the retro-orbital plexus of all animals into appropriate tubes containing EDTA 1:9 (v/v).

#### 2.7.5. Determination of Endogenous and Exogenous Coagulation Indexes

On day 14, all animals were anaesthetized with 6% pentobarbital sodium. After 40 min, the required blood samples were taken from abdominal aorta into appropriate tubes containing 3.8% sodium citrate 1: 9 (v/v). An aliquot of 1 mL blood sample of each animal was used for determination of the blood viscosity at high, medium and low shear rates. Remaining samples were centrifuged at 3000 rpm for 10 min to obtain plasma for the determination of TT, APTT, PT and FIB.

#### 2.7.6. Statistical Analysis

Data were expressed as mean ± standard deviation (SD) for each group. Statistical comparisons between groups were performed by one-way analysis of variance (ANOVA) using SPSS 17.0 software. Significance was accepted at* P*<0.05.

## 3. Results

### 3.1. ANOVA

The assay results of individual prepared slices from the 2x3 full factorial experiment are shown in Table 2 and the score results and ANOVA analyses are shown in Tables 3 and 4, respectively.

Note the following: divide the measured catalpol and verbascoside content values by the prespecified maximum value of 1 to separately calculate the score of each component, and the sum of two component scores is the composite score.

### 3.2. Validation of the Optimal Integrative Process and Comparison with Traditional Process

**Figure 1:**
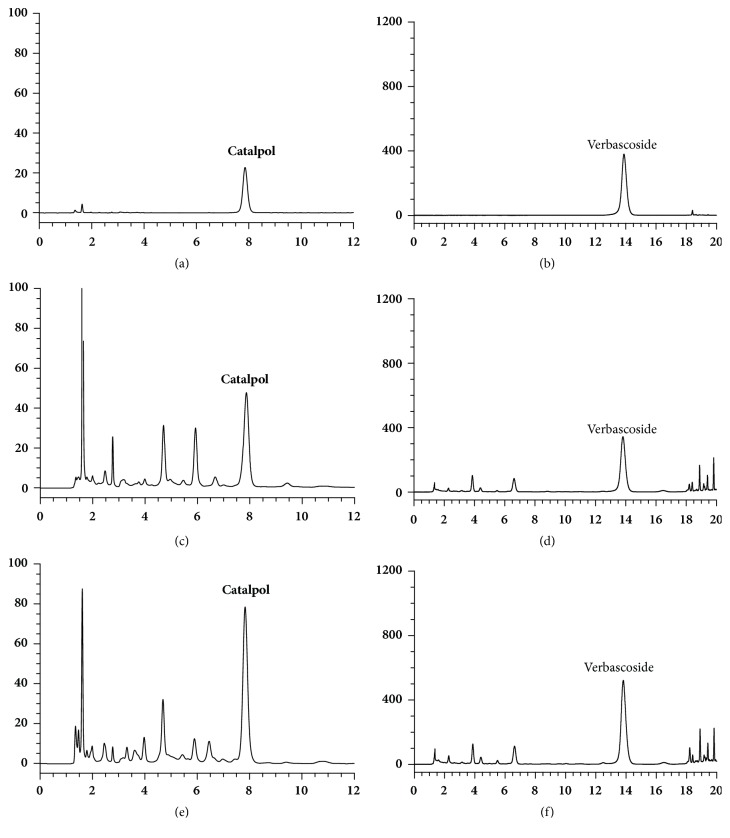
HPLC chromatograms of reference substances (a, b), traditional method-processed samples of Rehmanniae Radix (c, d), and Integrative method-processed samples of Rehmanniae Radix (e, f).

The mean contents of catalpol and verbascoside in the integrative method-processed samples were 4.30% and 0.33%, respectively, while the corresponding values for two components in traditional stepwise-processed samples were 2.61% and 0.21%, respectively. The typical HPLC chromatograms and the assay results of both prepared slices are shown in Figure 1 and Table 5.

### 3.3. Pharmacological Results

#### 3.3.1. Rectal Temperature

**Figure 2:**
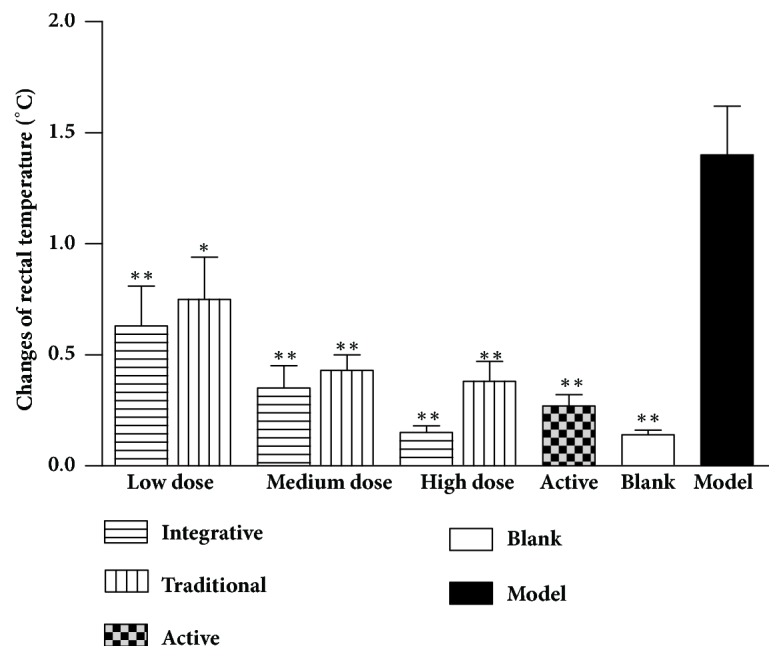
The changes in rectal temperature induced by Rehmanniae Radix obtained by two processing techniques in rats. Note that, *∗P*<0.01, and *∗∗P*<0.05 vs. the model group.

The differences in rectal temperature (Figure 2) in the dried ginger model group were significantly higher than the blank group rats (*P*<0.01). In the integrative groups and traditional groups, the extracts caused significant decreases in the rectal temperature with increased concentration compared to the model group animals (*P*<0.01,* P*<0.05).

#### 3.3.2. Hemorheological Measurements

**Figure 3:**
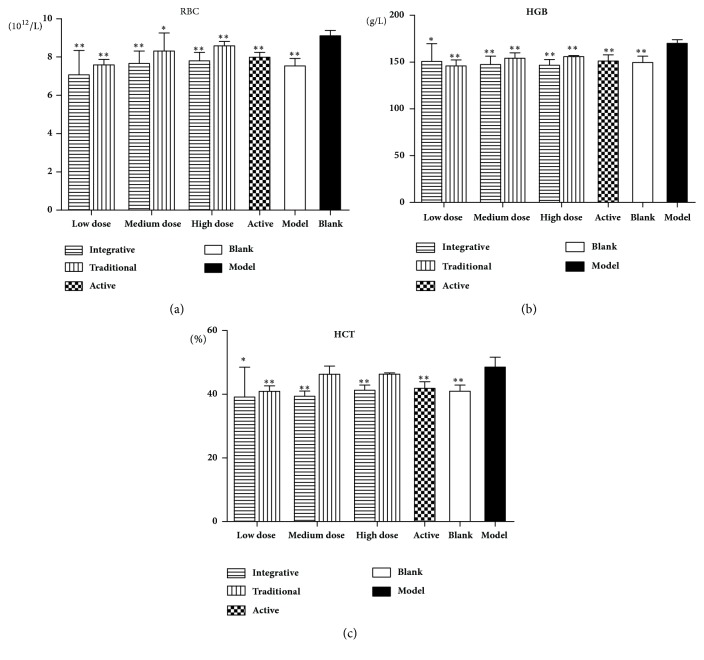
Effects of Rehmanniae Radix obtained by two processing techniques on red blood cell in rats. Note that, *∗P*<0.01, and *∗∗P*<0.05 vs. the model group. (a) RBC, (b): HGB, and (c) HCT.

**Figure 4:**
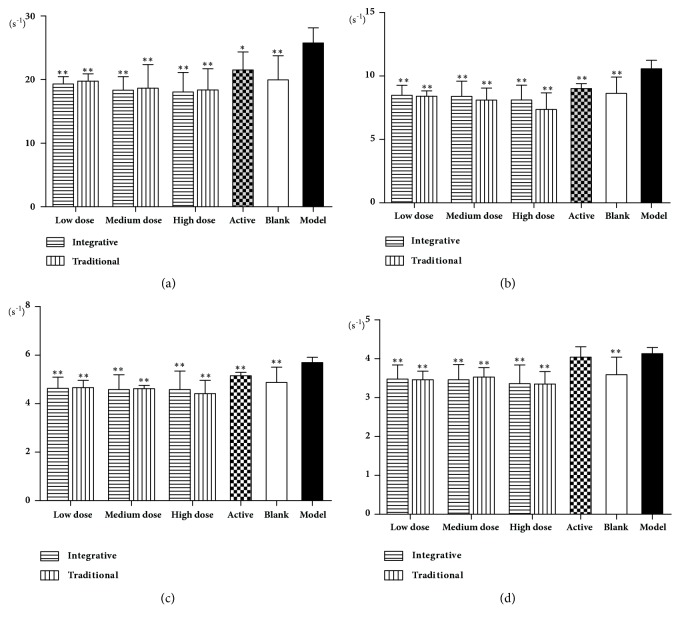
Effects of Rehmanniae Radix obtained by two processing techniques on blood viscosity in rats. Note that, *∗*P<0.01, and *∗∗*P<0.05 vs. model group. (a): 1, (b): 5, (c): 30, and (d): 200.

As shown in Figures 3 and 4, RBC, HGB, HCT, and blood viscosity at high, medium, and low shear rates were increased significantly in the model group rats, compared to the blank group animals (*P*<0.01). In the active group and all other treated groups, there were significant decreases in RBC, HGB, and HCT compared to the model group (*P*<0.01,* P*<0.05), as well as the blood viscosity at high, medium, and low shear rates (*P*<0.01,* P*<0.05).

#### 3.3.3. Determination of Endogenous and Exogenous Coagulation Indexes

**Figure 5:**
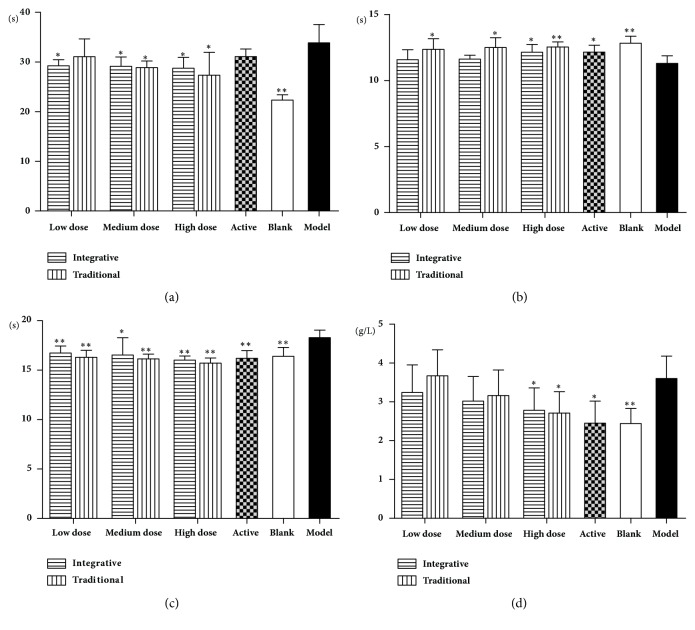
Effects of Rehmanniae Radix obtained by two processing techniques on the four blood coagulation indexes in rats. Note that, *∗P*<0.01, and *∗∗P*<0.05 vs. the model group. (a) TT, (b) PT, (c) APTT, and (d) FIB.

Compared to the blank group animals, there were significant increases in TT, APTT, and FIB, as well as a significant decrease in PT observed in rats of blood-heat and hemorrhage model induced by dried ginger (*P*<0.01). Compared to the model group, however, APTT and FIB values were decreased significantly, and PT value increased significantly in the active group animals (*P*<0.05,* P*<0.01). In all integrative and traditional groups, significant decreases in the TT and APTT values were consistently observed compared to the model group (*P*<0.05,* P*<0.01). For PT, there was a significant increase in the animals of the high-dose integrative group and all animals in the three traditional groups (*P*<0.05). At high dose level, both integrative and traditional method-processed products caused a significant decrease in FIB values (*P*<0.05). These results are given in Figure 5.

## 4. Discussion

According to ChP 2015, Rehmanniae roots are cut into thick slices to obtain Sheng Dihuang, and the thickness of 2-4 mm is used for General Quality Control Methods for Crude Drugs and Prepared Slices. Based on our observations that Rehmanniae roots were easy to break when cut into thin slices, while the roots were not easy to dry and lose moisture when cut into thicker slices, we selected the thickness as 5 mm and the slices of Rehmanniae roots met the requirements of ChP 2015 after drying.

The temperature setting in addition to the slice thickness may also be a key parameter in this experiment. The ANOVA results showed that factor A (drying temperature,* P*<0.05) had a highly significant effect on both catalpol and verbascoside in Rehmanniae Radix, but no similar effect was observed for factor B (drying time,* P*>0.05). The lowest level of drying at 50°C was selected as the best condition for factor A, i.e., A_1_. Furthermore, the contents of catalpol and verbascoside were significant higher after drying for 18 and 20 h at 50°C than drying for 16 h at 50°C. There is no significant difference observed for samples drying at 50°C for 18 h versus 20 h. The best combination of process conditions is A_1_B_2_, i.e., drying for 18 h at 50°C. As per ChP 2015, the contents of catalpol and verbascoside are required to be “not less than 0.20%” for catalpol and “not less than 0.020%” for verbascoside, respectively. With both processing methods, the contents of catalpol and verbascoside in the processed samples conformed to the compendial requirements. The results demonstrated that the optimized integrative process was robust and feasible. In addition, the contents of catalpol and verbascoside in the integrative processed samples were higher than the traditional stepwise-processed samples. We postulate this difference may be related to a reduction in the loss of active components during integrative process which avoids the repeated steps of drying and water-treatment.

As demonstrated by modern pharmacological experiments, ginger affects platelet aggregation and coagulation [21–25]. We have established the rat model of blood-heat and hemorrhage syndrome based on Traditional Chinese Medicine characteristics by ginger [26]. Given its known heat-clearing and blood-cooling effects, Rehmanniae Radix showed the ability to overcome the hot and spicy effect of dried ginger and consequently induced fluid depletion, improved blood viscosity, and reduced the elevated RBC count and hemoglobin concentration in our rat model of dried ginger-induced blood-heat and hemorrhage. The rectal temperature increased significantly in rats given with dried ginger extract, but this abnormal temperature changes induced by dried ginger were inhibited significantly with the active comparator (Yunnan Baiyao). In both integrative and traditional groups, Rehmanniae Radix at respective three dose levels showed the different degrees of improvement effects on hemorheological abnormalities observed in the blood-heat and hemorrhage rats. There were significant increases in TT, APTT, and FIB in the blood-heat and hemorrhage model rats. These coagulation parameters were shortened in rats given with two kind prepared slices. The results demonstrating integrative method-prepared slices seemed to be more pronounced with respect to the effect of clearing heat and cooling the blood, compared with traditional prepared slices at the same dose.

As we know, Chinese herbs are characterised by multiple ingredients, multiple targets, and multiple ways to exert effects. Although catalpol and verbascoside are widely recognized active ingredients in Rehmannia Radix, they cannot represent all the pharmacodynamics of Rehmannia Radix [17, 18, 27, 28]. Therefore, it is possible that there is not much pharmacological difference between the integrated method and the traditional method although the catalpol content is doubled by using the integrated method. These results indicate that the relationship between ingredients and pharmacodynamics of Rehmannia Radix during processing needs to be further studied.

## 5. Conclusions

An integrative processing technology of Rehmanniae Radix was optimized by full factorial experiment based on the contents of catalpol and verbascosid. Then the potential differences of the prepared slices obtained by integrative method versus traditional method were investigated by evaluating the hemostasis and hemorheological effects in the model rats. As compared with traditional prepared slices, the contents of catalpol and verbascoside were higher in the integrative processed samples and the hemostasis and hemorheological effects were more pronounced. Considering the active components and pharmacological effects, as well as the time and energy consumed, it can be concluded that the integrative processing technique is superior to the traditional processing technique. Namely, the integrative processing technique for Rehmanniae Radix may replace the traditional processing technique.

## Figures and Tables

**Table 1:** Factors and levels tested in the preliminary experiment.

Level	Factor	
	Temperature [°C] (A)	Time [h] (B)
1	50	16
2	60	18
3	70	20

**Table 2:** Assay results from factorial experiment(n=2).

Temperature [°C]	Time [h]	Catalpol [%]	Verbascoside [%]
50	16	3.802, 3.914	0.2801, 0.2946
50	18	4.195, 4.427	0.2950, 0.3267
50	20	4.259, 4.300	0.3063, 0.3204
60	16	3.933, 3.891	0.2506, 0.2396
60	18	3.700, 3.839	0.2344, 0.2381
60	20	3.611, 3.724	0.2480, 0.2777
70	16	3.968, 4.078	0.2291, 0.2548
70	18	3.898, 4.057	0.2501, 0.2213
70	20	3.690, 3.653	0.2381, 0.2385

**Table 3:** Score results.

Temperature [°C]	time [h]		
	16	18	20
50	1.716, 1.786	1.850, 2.000	1.900, 1.952
60	1.655, 1.612	1.569, 1.596	1.575, 1.691
70	1.598, 1.701	1.646, 1.594	1.562, 1.555

**Table 4:** ANOVA results.

Source	SS	df	MS	F	P-value	F crit
A	0.2592	2	0.13	39.21	3.6E-05	4.26
B	0.0035	2	0.0018	0.53	0.61	4.26
A × B	0.0491	4	0.012	3.72	0.047	3.63
SS_e_	0.0298	9	0.0033			
SS	0.3416	17				

**Table 5:** Comparison of integrative prepared slices with traditional prepared slices (n=3).

Processing technology	Catalpol [%]	Verbascoside [%]
Integrative method	4.30	0.33
Traditional method	2.61	0.21

## Data Availability

The data used to support the findings of this study are available from the corresponding author upon request.
